# Intergenerational Relationships in an Economically Vulnerable Community: Findings From the Flint Women’s Study

**DOI:** 10.1093/geroni/igaa057.1112

**Published:** 2020-12-16

**Authors:** Rodlescia Sneed

**Affiliations:** Michigan State University, Flint, Michigan, United States

## Abstract

Older women in economically disadvantaged communities often balance a range of relationships that present both benefits and challenges. The current study describes older women’s perceptions of the benefits and challenges of maintaining intergenerational relationships with younger women and children in their community. We used secondary data from the Flint Women’s Study, a qualitative interview project that included 60-90 minute structured interviews with 100 women who either lived or worked in the Flint, Michigan metropolitan area. Interviews were recorded, transcribed, and coded using the belongingness theory framework. Special populations (including older women) were coded in the dataset. The main benefits of intergenerational relationships included feeling valued, social connection, and giving back to future generations. In particular, intergenerational relationships allowed older women in this economically disadvantaged community to leave behind a meaningful social legacy in the absence of a meaningful economic legacy. Despite the perceived benefits, older women had difficulty effectively forming intergenerational relationships. Many reported being naturally isolated from those of younger generations. For those who were not isolated, many cited challenges related to grandparent caregiving, which was often connected to workforce conflicts, financial burden, caring for multiple generations well into adulthood, managing grandchildren’s traumas, and a lack of technological proficiency needed to meet grandchildren’s educational needs. Participants described the impact of these challenges on older women who lived in disadvantaged communities. Future programming should consider the needs of older women in disadvantaged communities and provide resources to maximize the benefits and minimize the challenges of intergenerational relationships in this population.

